# Complex Care in Dementia: Older Caregivers Provide More, Longer-Hour Caregivers Provide Less

**DOI:** 10.1093/geroni/igaf122.3905

**Published:** 2025-12-31

**Authors:** Sutthinee Thorngthip, Kylie Meyer

**Affiliations:** Case Western Reserve University, Cleveland, Ohio, United States; Case Western Reserve University, Cleveland, Ohio, United States

## Abstract

As the backbone of dementia care, family caregivers play an essential role in supporting individuals living with dementia as they provide a variety of care, from helping with daily activities (e.g., dressing) to assisting with complex care tasks (e.g., wound care, managing medication). Family caregivers often provide complex care without sufficient training, which can negatively affect their mental health and increase burden. However, few studies focus on characterizing complex care provided by family caregivers to inform such intervention. This study identified complex care tasks performed by caregivers and explored associations between the number of tasks provided and caregiver age, weekly hours of caregiving, and confidence with caregiving. A total of 131 caregivers completed surveys. Complex care questions were based on 17 items from the 2021 national AARP Home Alone report. Findings revealed that caregivers performed an average of 5.11 complex care tasks (SD = 2.54). The most frequent tasks were managing medications (93.9%, n = 123), preparing special diet (57.3%, n = 75), using telehealth equipment (56.5%, n = 74). Furthermore, older caregivers provided more complex care tasks (r= .215; p = .014), while caregivers providing a higher number of caregiving hours performed fewer tasks (r= .302; p<.001), potentially due to the integration of formal care support. Caregivers performing more complex care tasks reported lower confidence in taking care of different aspects of dementia care (r=.234; p= .007). The findings highlight the need for tailored caregiver training programs and accessible resources to equip family caregivers with skills and confidence for complex care tasks.

